# Unveiling the functional diversity of ionotropic glutamate receptors in the Pacific oyster (*Crassostrea gigas*) by systematic studies

**DOI:** 10.3389/fphys.2023.1280553

**Published:** 2023-10-25

**Authors:** Xueshu Zhang, Linfang Zhang, Yiran Si, Xue Wen, Lingling Wang, Linsheng Song

**Affiliations:** ^1^ Liaoning Key Laboratory of Marine Animal Immunology and Disease Control, Dalian Ocean University, Dalian, China; ^2^ Southern Laboratory of Ocean Science and Engineering, Zhuhai, Guangdong, China; ^3^ Liaoning Key Laboratory of Marine Animal Immunology, Dalian Ocean University, Dalian, China; ^4^ Dalian Key Laboratory of Aquatic Animal Disease Prevention and Control, Dalian Ocean University, Dalian, China

**Keywords:** ionotropic glutamate receptors, Crassostrea gigas, metal stress, environmental sensing, physiological adaptation

## Abstract

Ionotropic glutamate receptors (iGluRs), pivotal in mediating excitatory neurosignals within the central nervous system, are instrumental in environmental stress responses. In this investigation, 12 iGluRs identified in the Pacific oyster are herein designated as *Cg*iGluRs, and further categorized into three distinct subfamilies based on their transmembrane domains. Cross-species evolutionary analysis unveiled a high degree of conservation in the sequence and structural attributes of these *Cg*iGluRs. These receptors are ubiquitously distributed across various tissues, with pronounced expression in the oyster’s mantle, labial palps, and gills, underlining their integral role in the oyster’s environmental sensing mechanisms. Post the D-shaped larval stage, a marked upward trend in *Cg*iGluRs expression was observed, denoting their critical involvement in oyster development beyond this phase. Exposure to five metals—cadmium (Cd), copper (Cu), zinc (Zn), mercury (Hg), and lead (Pb)—elicited a significant upregulation of *Cg*GRIA4 expression, indicating a robust response to metal stress. A KEGG enrichment analysis on 142 genes, exhibiting parallel expression trends with *Cg*GRIA4 under metal stress, suggests that *Cg*GRIA4 could augment excitatory signal transmission by activating glutamatergic and dopaminergic synapses, thereby contributing to the metal stress response in the oyster. This inquiry not only bolsters our comprehension of the iGluRs gene family in metal stress response but also paves the way for future exploration of its cardinal role in cellular signaling and environmental adaptability.

## 1 Introduction

Glutamate, the chief excitatory neurotransmitter within the nervous system, directs a plethora of physiological functions, encompassing neural remodeling (Endo et al., 2021), environmental sensing (Wen et al., 2020), and signal transduction (Qiu et al., 2020), via a diverse array of receptors. Pharmacological classifications segregate glutamate receptors into two primary categories: ionotropic (iGluRs) and metabotropic (mGluRs) receptors (Zhu and Gouaux, 2017). A substantial body of research accentuates the superior velocity of iGluRs in information transmission relative to mGluRs, underscoring their capability for rapid environmental stress responses. iGluRs, functioning as multimeric ion channels, are tasked with the swift excitatory transmission in the nervous system. Upon binding to pre-synaptically released glutamate, iGluRs transduce signals into post-synaptic neuronal excitation within milliseconds. This complex process gives rise to synaptic currents, crucial for neural regulatory functions, and modulates perception and information transmission (Moretto et al., 2018). iGluRs are further classified into N-methyl-D-aspartate (NMDA) receptors, *α*-amino-3-hydroxy-5-methyl-4-isoxazolepropionic acid (AMPA) receptors, and kainate receptors (Mayer, 2016). According to the Motif structure diagram of model animals (human, mouse, zebrafish), we can see the differences among the three. Compared with NMDA-type receptors, Motif 8 and Motif 9 exist for AMPA-type receptors and KA-type receptors (Supplementary Figure S1). iGluRs subunits, dividing four modular structural domains including amino-terminal domain (ATD), ligand-binding domain (LBD), transmembrane domain (TMD), and C-terminal domain (CTD), coalesce into tetramers within their respective subclasses, forming ligand-gated ion channels (Karakas et al., 2015). The LBD contains two half-domains S1 and S2, which are closed to each other when LBD binds glutamate (Armstrong et al., 1998), and the Lig_Chan domain contains three transmembrane regions M1, M2, M3 and ion channel pore P (Kuner et al., 2003). Despite the extensive investigation of iGluRs in humans, mice, zebrafish, and other vertebrates over past decades (Herbrechter et al., 2021), due to their integral role in neuronal function, research in mollusks, particularly bivalves, is still nascent.

The rapid advancement of industry and agriculture in recent years has triggered a surge in marine pollution (Rahman et al., 2022). The environmental exposure to neurotoxic metals and metalloids, including cadmium, lead, mercury, copper, and zinc, has escalated into a global health concern, affecting millions worldwide (Liu et al., 2023). Research suggests that environmental neurotoxic metal stress can compromise neurotransmitter receptor function, thereby impinging on neural development, behavior, cognition, and precipitating neurodegeneration (Carmona et al., 2021). Existing evidence implicates Cd in directly affecting synaptic transmission mediated by AMPA receptors (Wang et al., 2008). Conversely, neurotoxic Pb exhibits significant selectivity for NMDA receptors, suggesting that the neurotoxicity of this metal is mediated by receptor-type-specific regulation (Marchetti and Gavazzo, 2003). Moreover, copper can bidirectionally modulate hippocampal neuronal synaptic activity: acute copper stimulation can impede signal transmission, but after a 3-h continuous copper stimulation, it amplifies the frequency and amplitude of AMPA currents (Peters et al., 2011). Recent research reveals that Cd downregulates NMDA receptors (GRIN2A and GRIN2B) and inhibits the activity of inhibitory glutamate receptor GluR2, while upregulating the phosphorylation of excitatory glutamate receptor GluR1, inducing functional impairment of glutamate receptors (Yang et al., 2023). Consequently, environmental neurotoxic metals can obstruct various functions of the entire nervous system via iGluRs, thereby disrupting organismal homeostasis (Pochwat et al., 2015). The chosen metals (Zn, Cu, Cd, Hg, and Pb), being prevalent marine pollutants with known iGluR interactions, are pivotal for examining environmental stress responses in Pacific oyster.

In this context, bivalves, such as the Pacific oyster (*Crassostrea gigas*), have emerged as a research focal point due to their unique resilience to metal pollution. Intriguingly, the oysters harbor high concentrations of metals without manifest toxicity, suggesting the evolution of sophisticated metal accumulation regulatory mechanisms (Jonathan et al., 2017). Investigations in vertebrates demonstrate the toxic effects of metals on ionotropic glutamate receptors, which can severely perturb iGluRs signal transmission (Sadiq et al., 2012). Prior research has corroborated the presence of a relatively comprehensive neuroendocrine system in oysters (Liu et al., 2018; Wang, 2022), yet reports on iGluRs and their regulation of metal ions are scant. Elucidating the mechanisms and strategies of bivalve iGluRs in response to metal stimulation holds profound implications for addressing environmental pollution and seafood safety issues.

Against this backdrop, the present study identified and systematically analyzed the iGluRs of *C. gigas*. Subsequently, the spatiotemporal expression spectrum of *Cg*iGluRs genes was scrutinized using the RNA-seq dataset. Furthermore, this study probed the expression level and characteristics of *Cg*iGluRs genes in the gills to decipher the molecular mechanisms underpinning oyster responses to heavy metal stress.

## 2 Materials and methods

### 2.1 Identification and characterization of iGluRs genes in *C. gigas*

The BLASTP tool was deployed to decipher the gene sequence of iGluRs in the Pacific oyster. Amino acid sequences of iGluRs from a broad spectrum of invertebrates and vertebrates were leveraged as queries against the NCBI^1^ and Uniprot databases^2^ (UniProt Consortium, 2018). This exhaustive search spanned species from sea hare to human, inclusive of *Xenopus tropicalis*, *Danio rerio*, and *Homo sapiens*. The oyster transcriptome and whole genome sequences were meticulously examined to identify candidate iGluRs genes. Subsequent analyses involved predicting amino acid sequences using the ORF Finder tool^3^, identifying conserved structural domains via the SMART program^4^ (Letunic and Bork, 2018), and detecting conserved motifs using the MEME Suite^5^ (Nystrom and McKay, 2021), with a maximum motif limit set to 12 (prevent motif overlap and maintain analysis accuracy). All results were visualized using TBtools (Chen et al., 2020). The Compute pl/Mw tool^6^ (Wilkins et al., 1999) was utilized to calculate the GRAVY (Grand average of hydropathicity), theoretical isoelectric point (pI), and molecular weight (Mw) of the pore domain, while the secondary structure was predicted using Geneious7.0.6^7^ (Kearse et al., 2012).

### 2.2 Phylogenetic analysis and chromosomal localization of iGluRs in *C. gigas*

For the phylogenetic analysis, iGluRs proteins from *C. gigas* and other selected species, including the invertebrates and vertebrates, were selected. The iGluRs amino acid sequences from selected species were retrieved from the NCBI and Uniprot databases (Supplementary Table S1). Multiple sequence alignment was executed using AliView software (Larsson, 2014), followed by the construction of an evolutionary tree based on the maximum likelihood method via PhyML (v3.0) software^8^ (Guindon et al., 2010). The tree was subsequently refined using FigTree (v1.4.4) software^9^. Chromosomal locations and sizes of the iGluRs genes in the oyster were derived from the oyster genome data (cgigas_uk_roslin_v1) (Peñaloza et al., 2021), analyzed through TBtools.

### 2.3 Spatiotemporal expression profiling of iGluRs in *C. gigas*

Expression analysis was conducted using the RPKM (Reads Per Kilobase Million) values of each iGluRs gene from the publicly available RNA-seq dataset of the oyster. This dataset spans various developmental stages and adult tissues. Expression patterns of these genes across different stages and tissues were visualized using a heatmap generated by TBtools.

### 2.4 Transcriptional response of iGluRs in *C. gigas* to heavy metal exposures

In investigating the transcriptional dynamics of iGluRs in oysters under heavy metal exposure conditions, we utilized an RNA-Seq dataset (Zhang et al., 2012), encompassing exposure data for Zn, Cu, Cd, Hg, and Pb. Specifically, oysters were exposed to one of the five metals (Zinc 1 mg/L, Cadmium 100 μg/L, Copper 100 μg/L, Lead 500 μg/L, Mercury 20 μg/L), with a control group subjected to seawater treatment. The concentrations of these metals were non-lethal, and no fatalities occurred during the experiment. The sampling time points were at 12 h and 9 days post-exposure. The original RNA-Seq data (Project number: PRJNA146329) were obtained from the NCBI database. Subsequently, these data were aligned to the oyster genome utilizing HISAT2 (v2.0.5) with default parameters. Gene expression levels were then estimated employing the Fragments Per Kilobase Million (FPKM) method. Temporal trends of gene expression under different metal exposures were analyzed and clustered using the Mfuzz R package (Kumar and Futschik, 2007) in R (version 4.2.3). A Venn diagram depicting the common expression trends of *Cg*GRIA4 under five metal exposures was constructed using jvenn^10^ (Bardou et al., 2014).

### 2.5 Pathway enrichment and interaction analysis of iGluRs in *C. gigas*

Following the Venn diagram analysis, KEGG enrichment analysis was performed on all intersecting treatments using the R package clusterProfiler (Yu et al., 2012). The enrichKEGG function was used to identify enriched KEGG pathways among the genes listed in the Venn diagram, with a *p*-value <0.05 set as the threshold for significance. To further elucidate the response mechanism of *Cg*GRIA4 to heavy metals, significantly enriched pathways (*p* < 0.05) involving the *Cg*GRIA4 gene were screened. A network diagram of these pathways was constructed using the KEGG network tool of OmicShare Tools^11^. Enrichment pathways and gene information are detailed in Supplementary Table S2. Collectively, through KEGG enrichment analysis and pathway network diagramming, we aim to gain a deeper understanding of the expression pattern of *Cg*GRIA4 under different metal exposures and its role in biological processes.

## 3 Result

### 3.1 Identification and characterization of iGluRs genes in *C. gigas*

To elucidate the genomic landscape of the oyster, a comprehensive analysis of the transcriptome and genome databases was undertaken, leading to the discovery of 12 iGluRs genes. These genes, detailed in Table 2, were classified into three distinct subfamilies based on sequence homology and domain architecture: AMPA receptors, NMDA receptors, and kainate receptors (Table 1). The open reading frames (ORFs) of *Cg*iGluRs spanned from 2,385 to 3,675, encoding between 794 and 1,224 amino acids. GRIA2 was found to be the most complex, comprising 19 exons and 18 introns (Supplementary Figure S2; Table 2). The predicted molecular weights of *Cg*iGluRs ranged from 89.49 to 138.79 kDa, with predicted isoelectric points (pI) between 5.78 and 8.81. The secondary structure of the proteins encoded by iGluRs suggested a composition of 29–53 alpha helices, 44 to 74 beta strands, 58 to 93 coils, and 52 to 99 turns (Table 2). The amino acid consistency between *Cg*iGluRs and iGluRs of other invertebrates ranged from 27.25% to 90.46%, and it ranged from 23.96% to 50.76% with vertebrate iGluRs (Table 3).

**TABLE 1:** Statistical table of gene members of iGluRs subfamily in different species.

Species	NMDAR	AMPAR	KAR	Total
*Homo sapiens*	7	4	5	16
*Mus musculus*	7	4	5	16
*Gallus gallus*	6	4	4	14
*Larimichthys crocea*	7	4	5	16
*Xenopus tropicalis*	7	4	5	16
*Danio rerio*	8	4	5	17
*Octopus bimaculoides*	3	0	1	4
*Aplysia californica*	1	1	3	5
*Lingula anatina*	2	0	1	3
*Strongylocentrotus purpuratus*	0	1	1	2
*Ciona intestinalis*	1	3	1	5
*Crassostrea virginica*	3	0	2	5

**TABLE 2:** Sequence characteristics of iGluRs gene family of *C. gigas*.

Gene name	Gene ID	cDNA length (bp)	ORF length (bp)	Exons no.	Introns no.	Amino acid no.	Molecular weight (kDa)	Theoretical PI	AlpHa no.	Beta no.	Colins no.	Turn no.	GRAVY of PD
GRIN3A	LOC105318495	4,665	3,675	9	8	1224	138789.05	7.04	53	74	93	99	−0.271
GRIK3	LOC105323215	3,257	2,595	3	2	864	97918.54	6.37	44	44	58	52	−0.206
GRIA2	LOC105336269	3,751	2,907	19	18	968	110793.88	8.35	38	54	68	68	−0.235
GRIK2-like	LOC105332320	4,499	2,751	15	14	916	104746.63	5.78	45	56	68	74	−0.226
GRIN2B	LOC105347230	5,380	3,357	17	16	1,118	129258.96	8.69	50	64	83	89	−0.280
GRIK1	LOC105348088	3,070	2,403	13	12	800	89486.81	6.15	36	47	60	55	0.025
GRIA4	LOC105327395	2,970	2,463	14	13	820	94127.40	6.68	37	48	59	59	−0.152
GRIK5	LOC105327397	4,422	2,385	12	11	794	90691.87	6.29	29	52	66	62	−0.116
GRIA1	LOC105326127	3,359	2,691	17	16	864	98015.03	8.81	42	57	68	71	−0.175
GRIA1-like	LOC105326132	3,141	2,640	17	16	879	100006.41	6.41	34	57	70	65	−0.204
GRIN2A	LOC105322565	3,407	2,529	14	13	842	95416.69	6.17	38	47	65	67	−0.339
GRIN1	LOC105333721	4,314	2,667	18	17	888	99428.99	6.13	52	51	65	59	−0.200

**TABLE 3:** Percentage of Identity(I) of *C.gigas* iGluRs with selected iGluRs proteins in other species.

Gene	*H. sapiens* (%)	*M. musculus* (%)	*G gallus*	*X tropicalis* (%)	*D. rerio* (%)	*L crocea* (%)	*C intestinalis*	*O bimaculoides*	*A californica*	*L anatina*	*D melanogaste*	*C*. *virginica*
GRIN3A	27.79	27.56	30.05%	28.16	26.28	29.34	—	43.72%	—	—	—	75.55%
GRIK3	28.79	28.92	28.69%	27.43	32.61	28.59	—	—	—	—	—	76.85%
GRIA2	42.64	42.52	44.19%	41.06	40.94	44.66	27.90%		39.74%	—	—	—
GRIK2-like	40.26	40.26	41.49%	40.60	40.76	40.52	27.25%	27.25%	45.16%	39.52%	32.39%	28.81%
GRIN2B	30.32	30.55	30.89%	30.89	30.48	34.72	—	—	—	39.64%	—	42.62%
GRIK1	24.00	24.05	24.65%	24.94	23.96	24.59	—	—	—	—	—	—
GRIA4	32.26	31.86	32.13%	31.77	31.51	37.88	—	—	—	—	—	—
GRIK5	31.08	31.58	—	34.65	33.07	30.33	—	—	23.79%	—	—	—
GRIA1	39.88	40.02	40.27%	40.62	39.21	38.86	—	—	—	—	39.42%	—
GRIA1-like	41.60	41.53	42.10%	42.54	40.54	38.98	—	—	—	—	42.76%	—
GRIN2A	33.14	33.14	33.90%	33.48	33.24	31.93	35.67%	53.42%	—	—	—	—
GRIN1	48.98	48.75	50.76%	48.29	47.07	47.65	34.97%	58.58%	—	58.24%	51.70%	90.46%

**FIGURE 1:**
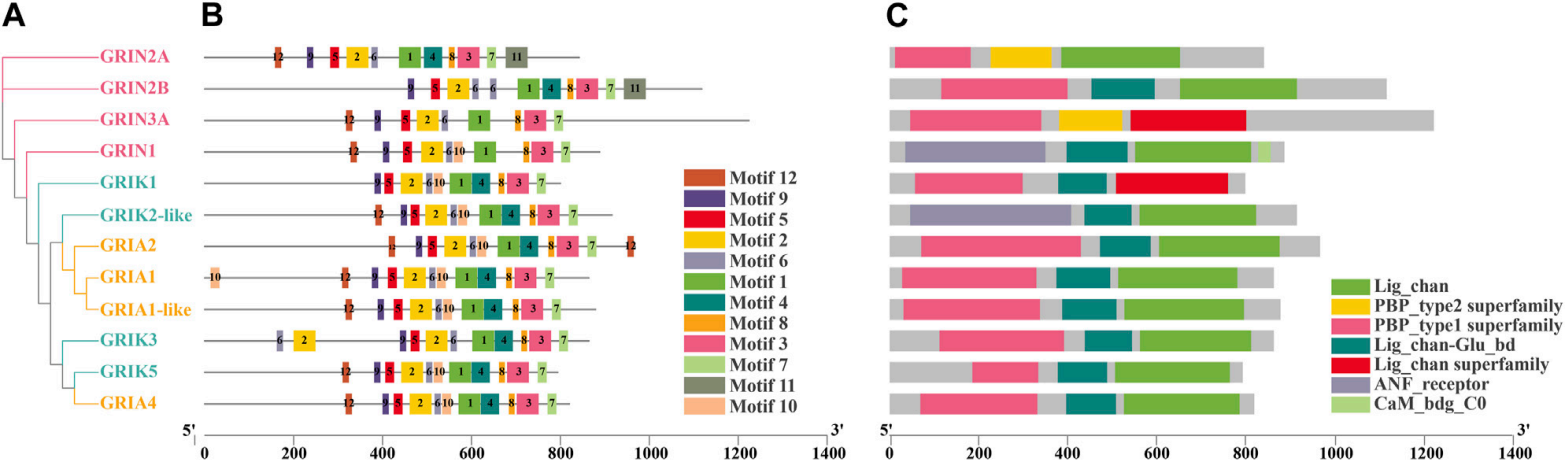
Phylogenetic relationships, protein motifs, and gene structures of 12 *Cg*iGluRs genes. **(A)** Phylogenetic tree of *Cg*iGluRs. Protein sequences were aligned using AliView, and the phylogenetic tree was constructed applying the maximum likelihood method. **(B)** Protein motifs of *Cg*iGluRs. Conserved motifs (1–12) are depicted by different colored boxes, with non-conserved sequences represented by black lines. Motifs were visualized using Tbtools. **(C)** Gene structures: Lig_Chan domains, PBP_type1 superfamily domains, Lig_chan superfamily domains, ANF_receptor domains, and PBP_Type_2 superfamily domains are represented by green, yellow, pink, blue, and red boxes respectively.

A phylogenetic tree was constructed for *Cg*iGluRs, and subsequent analysis of domain information and gene base sequence was conducted (Figure 1). All *Cg*iGluRs were found to possess a Pfam Lig_Chan domain centrally, which belongs to the TMD module. (Figure 1C). The N-terminus of the *Cg*NMDA subfamily was found to feature a PBP_type1 superfamily domain, which belongs to the LBD module. Certain *Cg*iGluRs also contained specific structural regions, such as the Cam_bdg_C0 domain at the C-terminus of *Cg*GRIN1, which is the key with NMDA-type receptors that allow calcium ions to pass through (Figure 1C). Twelve conserved motifs were identified in *Cg*iGluRs, with *Cg*iGluRs sharing eight common motifs (1–3, 5–9; Figure 1B). *Cg*GRIN contained a unique motif, motif 11. All proteins, except for *Cg*GRIN3A and *Cg*GRIN1, possess motif 4 (Figure 1B). *Cg*GRIN2B and *Cg*GRIK3 lack motifs 10 and 12, *Cg*GRIN2A and *Cg*GRIN3A lack motif 10, and *Cg*GRIK1 lacks motif 12 (Figure 1B).

### 3.2 Phylogenetic relationship and Chromosomal Localization of *Cg*iGluRs

**FIGURE 2:**
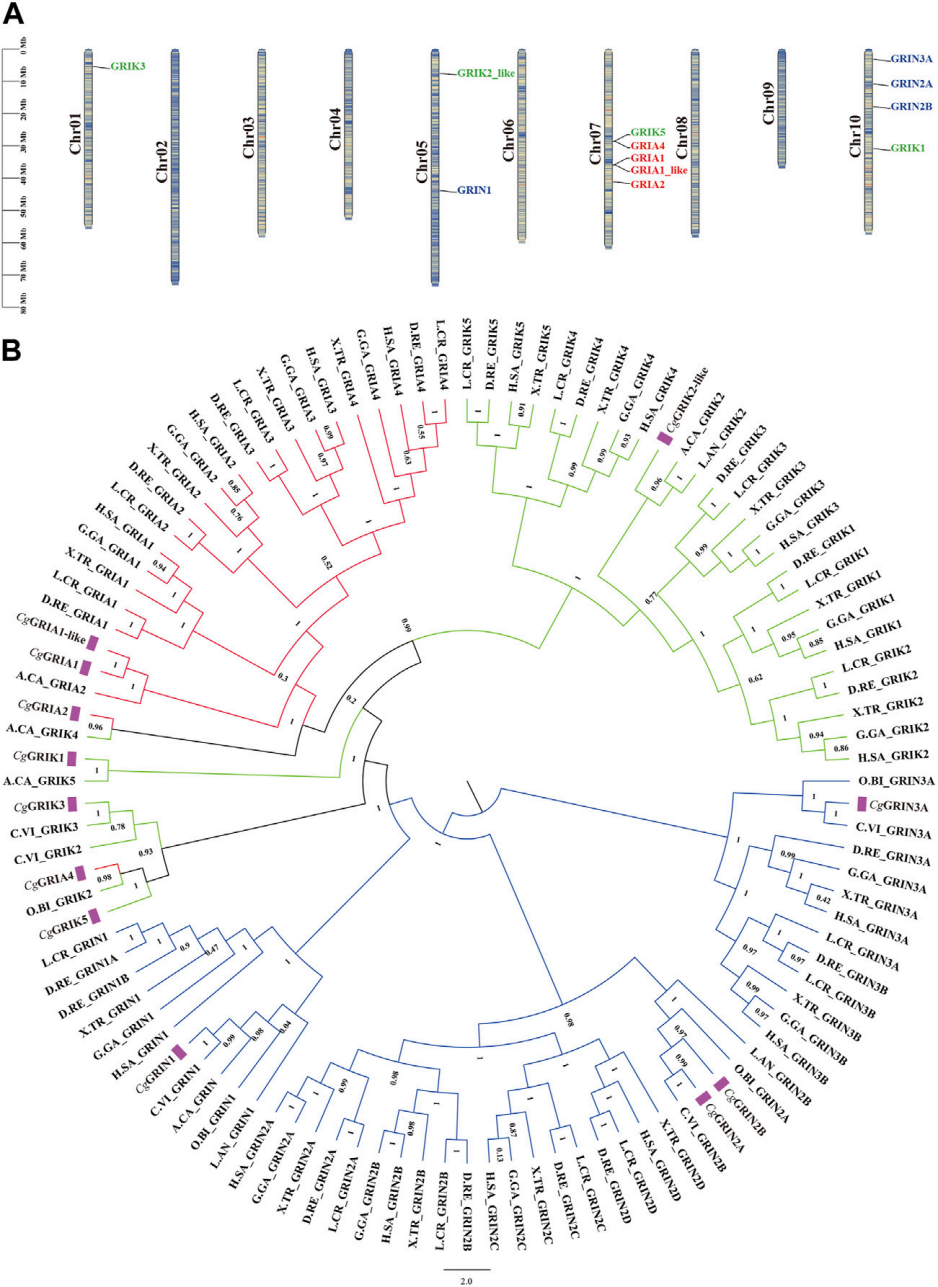
Phylogenetic Analysis and Chromosomal Localization of *Cg*iGluRs. **(A)** Chromosomal distribution of the 12 *Cg*iGluRs genes, along with the dispersion of duplicate gene pairs. Each gene is positioned on a chromosome according to its physical location, with the chromosome number (Chr01-Chr10) indicated on the left. **(B)** A multispecies phylogenetic tree, derived from the protein sequences of iGluRs from *C. gigas* and other selected species, was constructed using the maximum likelihood method and supported by 1,000 bootstrapped pseudoreplicates. *Cg*iGluRs are marked in purple. Branches representing different subfamilies are highlighted in distinct colors (*Cg*NMDAR: blue, *Cg*AMPAR: red, *Cg*KAR: green). Species abbreviations are as follows: Cg, *Crassostrea gigas*; H.SA, *Homo sapiens*; G.GA, *Gallus gallus*; X. TR, *Xenopus tropicalis*; D.RE, *Danio rerio*; O.BI, *Octopus bimaculoides*; A.CA, *Aplysia californica*; L. AN, *Lingula anatine*; L. CR, *Larimichthys crocea*; C.VI, *Crassostrea virginica*.

A chromosome map of *Cg*iGluRs was constructed based on the oyster genome sequence (Figure 2A). All 12 identified *Cg*iGluRs were found to be located on the oyster chromosomes, primarily on chromosomes 1, 5, 7, and 10. Chr 7 hosts the majority (5 *Cg*iGluRs) of *Cg*iGluRs genes, while Chr 5 contains only two. Most *Cg*iGluRs genes are found on Chr7 and Chr10 (9 out of 12, 75%), suggesting that the number of *Cg*iGluRs genes is not related to chromosome size (Figure 2A).

In this study, a phylogenetic tree was constructed by comparing the full-length amino acid sequences of *Cg*iGluRs and those from other species (Figure 2B; Table 4). The results reveal that the *Cg*iGluRs family can be divided into two main branches: NMDA and non-NMDA receptors, which are further subdivided into three subfamilies, namely, GRIA, GRIK, and GRIN (Figure 2B). The classification of each subfamily is based on genetic similarity. Within each subfamily, the iGluRs members of vertebrates and invertebrates form independent branches. Notably, the genes of the American oyster and the oyster share the closest evolutionary relationship within the same iGluRs subfamily (Figure 2B). In the specific construction of the phylogenetic tree, the red branch represents the GRIA subfamily. Among them, *Cg*GRIA1, *Cg*GRIA1-like, and A.CA GRIA2 form a branch. *Cg*GRIA2 and A.CA GRIK4 form a branch. *Cg*GRIA4, O.BI GRIK2, and *Cg*GRIK5 form a branch, and then form a branch with *Cg*GRIK3, C.VI GRIK2, and C.VI GRIK3 forms a branch. The green branch represents the GRIK subfamily. In this subfamily, *Cg*GRIK1 forms a branch with the A.CA GRIK5, while *Cg*GRIK2-like forms a branch with A.CA GRIK2, L. AN GRIK2. The blue branch represents the GRIN subfamily, which contains four *Cg*GRIN genes. It is worth noting that the number of three subfamilies including GRIA, GRIK, and GRIN has significantly increased in vertebrates, indicating that these iGluRs subfamilies have been continuously expanded during evolution (Figure 2B).

**TABLE 4:** Comparison table of scientific name of species.

Abbreviation	Scientific name
H.SA	*Homo sapiens*
G.GA	*Gallus gallus*
X.TR	*Xenopus tropicalis*
D.RE	*Danio rerio*
O.BI	*Octopus bimaculoides*
A.CA	*Aplysia californica*
L.AN	*Lingula anatina*
L.CR	*Larimichthys crocea*
C.VI	*Crassostrea virginica*
Cg	*Crassostrea gigas*

### 3.3 Spatiotemporal Expression Patterns of *Cg*iGluRs

**FIGURE 3:**
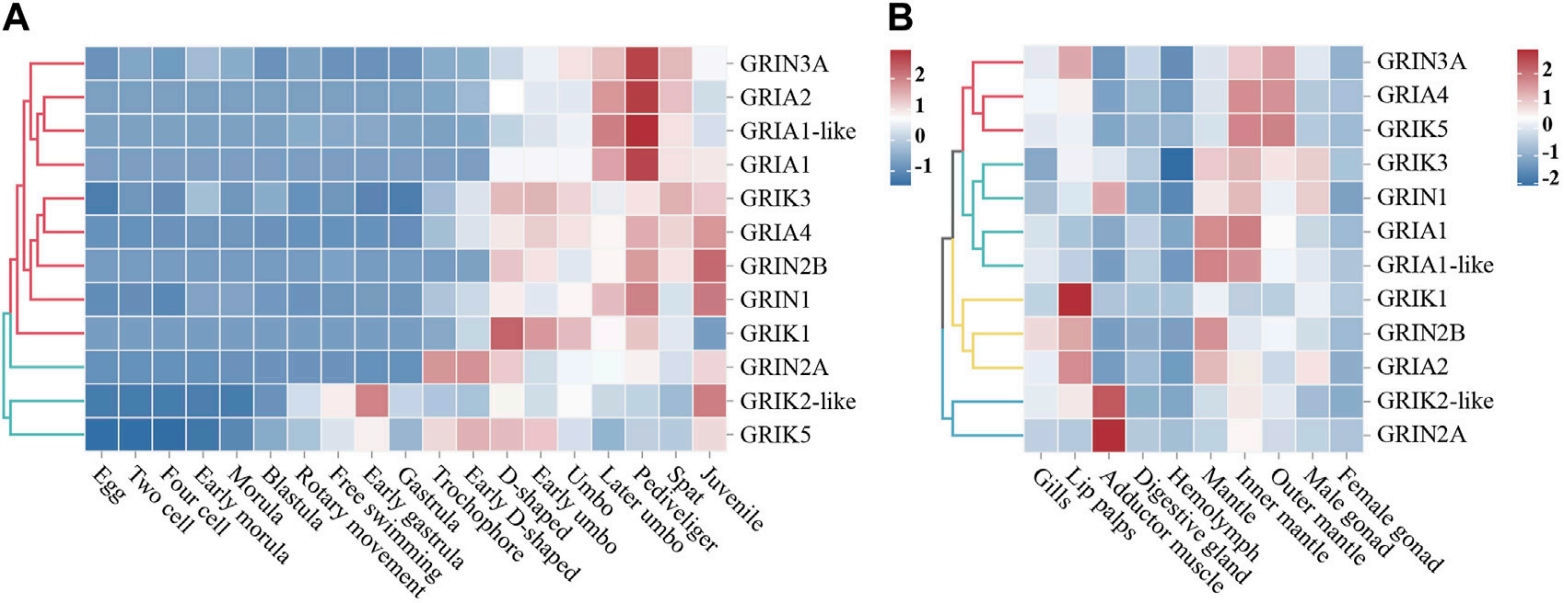
Spatiotemporal Expression Patterns of *Cg*iGluRs. **(A)** A heat map depicting the expression profiles of *Cg*iGluRs genes across various developmental stages, quantified by Log_10_RPKM. The stages included are egg, two-cell, four-cell, early morula, morula, blastula, rotary movement, free swimming, early gastrula, gastrula, trochophore, early D-shaped, D-shaped, early umbo, umbo, later umbo, pediveliger, spat, and juvenile. Based on the expression patterns of *Cg*iGluRs, two primary clusters are delineated, represented in red and blue colors. **(B)** A heat map presenting the tissue-specific expression profiles of *Cg*iGluRs genes in adult oysters, quantified by Log_10_RPKM. The tissues represented include gills, lip palps, adductor muscle, digestive gland, hemolymph, mantle, inner margin of mantle, outer margin of mantle, female gonad, and male gonad. Based on the expression patterns of *Cg*iGluRs are grouped into four clusters, denoted by red, cyan, yellow, and blue.

RNA-seq datasets from different developmental stages and adult tissues of the oyster were used to detect the spatiotemporal expression spectrum of *Cg*iGluRs (Figure 3). The expression patterns of *Cg*iGluRs can be divided into two groups across different developmental stages (Figure 3A). The first group consists of 9 *Cg*iGluRs that are highly expressed after D-shaped larvae, and these *Cg*iGluRs have higher expression levels during the Pediveliger period than during the Later umbo larva period and Spat period. The second group consists of 3 *Cg*iGluRs that are highly expressed before D-shaped larvae, and these *Cg*iGluRs have different expression patterns throughout the development of the oyster.

In adult oyster tissues, *Cg*iGluRs expression patterns are categorized into four distinct groups as illustrated in Figure 3B. GRIN3A, GRIA4, and GRIK5 from the first group predominantly exhibit expression in the mantle and its edge. The second group, which includes *Cg*GRIK3, *Cg*GRIN1, *Cg*GRIA1, and *Cg*GRIA1-like, primarily shows expression in the inner edge of the mantle. The labial palps are the main expression site for the third group, containing *Cg*GRIK1, *Cg*GRIN2B, and *Cg*GRIA2. The adductor muscle expresses the fourth group, represented by *Cg*GRIK2-like and *Cg*GRIN2A. Additionally, peak expressions of *Cg*iGluRs in adult oysters are found in neural tissues associated with environmental perception, encompassing areas like the labial palps, adductor muscle, and mantle edges.

### 3.4 *Cg*iGluRs expression under metal exposures

**FIGURE 4:**
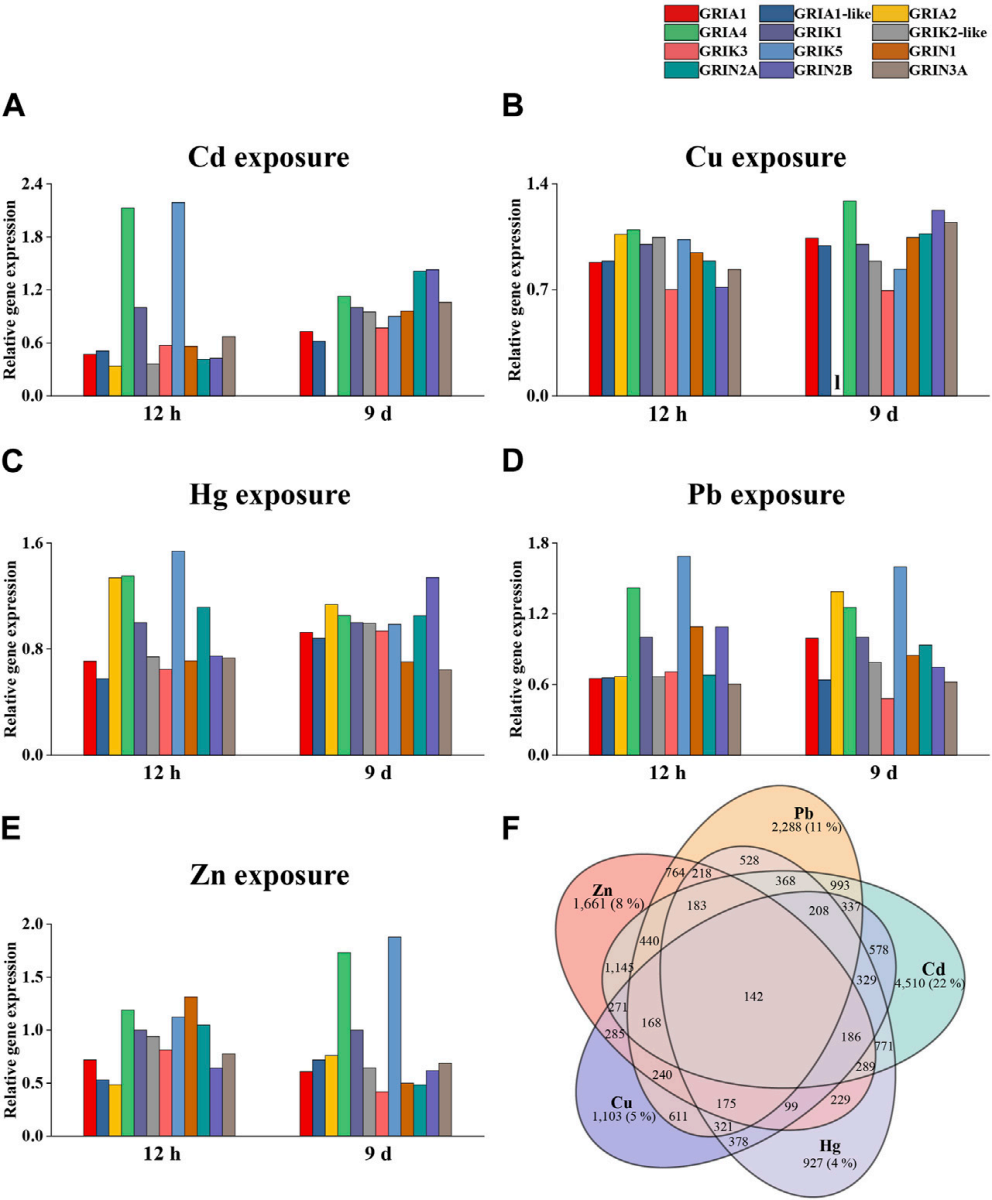
Expression of *Cg*iGluRs in Response to Metal Exposure. **(A)** Changes in relative gene expression (expressed as fold change relative to control) in oysters exposed to cadmium (Cd). **(B)** Relative gene expression alterations (expressed as fold change relative to control) in oysters in response to copper (Cu) exposure. **(C)** Modulations in relative gene expression (expressed as fold change relative to control) in oysters following Mercury (Hg) exposure. **(D)** Adjustments in relative gene expression (expressed as fold change relative to control) in oysters subjected to lead (Pb) exposure. **(E)** Changes in relative gene expression (expressed as fold change relative to control) in oysters upon zinc (Zn) exposure. **(F)** A Venn diagram presenting the genes demonstrating similar expression trends to GRIA4 under the stimulation of the five metals.

To detect the expression pattern of *Cg*iGluRs in response to heavy metal stress, the RNA-seq dataset of oyster gills under the stress of five heavy metals Zn, Cu, Cd, Hg, Pb were analyzed (Figure 4). The expression levels of GRIA1 and GRIA1-like mRNA decreased after short-term exposure to the five heavy metals, but under long-term exposure to Cu, the expression of these two *Cg*iGluRs returned to normal level (Figures 4A–E). In addition, long-term exposure to Cu and Cd inhibited the expression of *Cg*GRIA2 (Figures 4A, B). Under short-term exposure to Cu, Cd, Hg, and Pb, the expression of *Cg*GRIA4 and *Cg*GRIK5 was upregulated (Figures 4A–D). The expression levels of *Cg*GRIA4 and *Cg*GRIK5 were upregulated under long-term exposure to Zn (Figure 4E). Collectively, the results highlight the pronounced responsiveness of *Cg*GRIA4 and *Cg*GRIK5 to heavy metal perturbations.

### 3.5 Mechanism of *Cg*GRIA4 in response to metal stress

**FIGURE 5:**
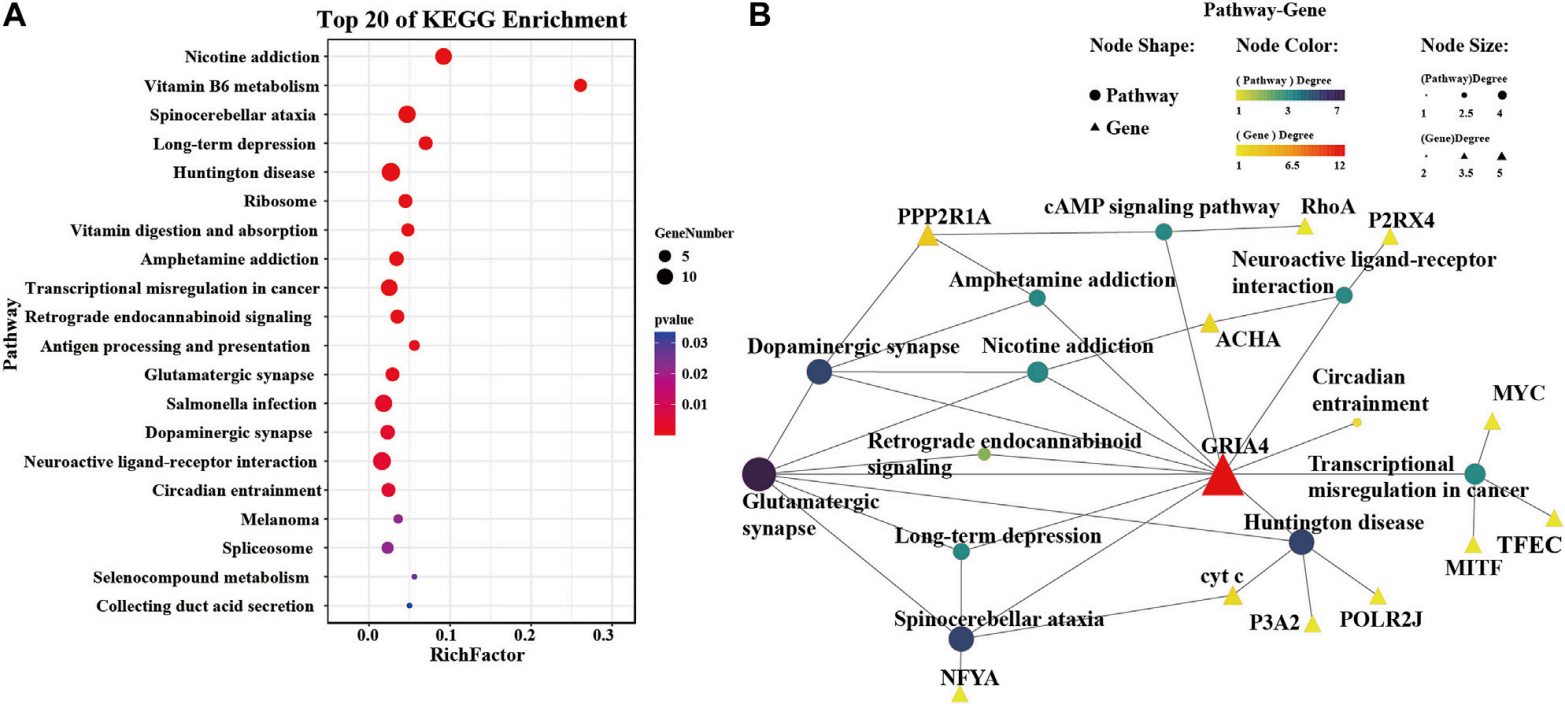
KEGG enrichment analysis of genes expressing similarly to *Cg*GRIA4. **(A)** The top 20 enriched KEGG terms identified among the 142 genes depicted in Figure 4F. **(B)** A network interaction model constructed from the KEGG enrichment analysis performed on the 142 intersecting genes presented in Figure 4F. This analysis enabled the identification of KEGG pathways significantly engaged by *Cg*GRIA4, and these pathways are depicted in the model to demonstrate their interconnections.

The analysis prioritized CgGRIA4 over GRIK5 due to the established association of AMPA-type receptors, to which GRIA4 belongs, with calcium ion permeation critical in metal stress response. To better understand GRIA4’s role in metal exposure, a detailed analysis following exposure to five metals identified 142 genes with similar expression trends (Supplementary Figure S3; Figure 4F). Through KEGG enrichment analysis of these genes, we found that pathways related to neural signal transmission, such as Glutamatergic synapse (ko04724), Dopaminergic synapse (ko04728), and Neuroactive ligand-receptor interaction (ko04080), were significantly enriched (Figure 5A). In addition, some antioxidant-related metabolic pathways, such as Vitamin B6 metabolism (ko00750), Vitamin digestion and absorption (ko04977), and Selenocompound metabolism (ko00450), were also significantly enriched (Figure 5A). To reveal the mechanism of *Cg*GRIA4 in metal stress response more deeply, we drew a KEGG network map of the genes in the 142 genes that share the same pathway with *Cg*GRIA4 (Figure 5B). In the KEGG network map, *Cg*GRIA4 mainly participates in the activation of Glutamatergic synapse and Dopaminergic synapse, and *Cg*GRIA4 mainly affects two neurodegenerative disease-related pathways, Spinocerebellar ataxia (ko05017), and Huntington disease (ko05016), and the activation of these two pathways is closely related to calcium ion homeostasis imbalance (Figure 5) (Begum et al., 2018; Zhou et al., 2018).

## 4 Discussion

Ionotropic Glutamate Receptors (iGluRs) are a key type of ion channel widely distributed across the animal kingdom. Upon activation, they transmit signals of sodium, potassium, or calcium ions, participating in various sensory processes (Manookin et al., 2008; Sánchez-Alcañiz et al., 2018) and playing a crucial role in physiological processes such as neuroplasticity (Budreck et al., 2013), learning and memory, cell life cycle, and immune defense. In this study, a complete set of iGluRs family genes was identified in the genome of the oyster *C. gigas*, and their protein structure, phylogenetic relationships, expression patterns during developmental stages and in the adult tissues under heavy metal stress were analyzed. The results provide a new perspective for a deeper understanding of the molecular evolution and functional diversity of the iGluRs channel family.

Our comprehensive genomic screening revealed the presence of 12 *Cg*iGluRs family genes in oysters. These genes are widely distributed across the three iGluRs subfamilies: NMDA receptors (NMDAR), AMPA receptors (AMPAR), and kainate receptors (KAR). By contrast, the iGluRs family gene combination in vertebrates, such as humans (Hansen et al., 2021), is more diverse, encompassing 7 NMDARs, 4 AMPARs, and 5 KARs. In vertebrate evolution, the expansion of KAR and AMPAR genes is notably more significant than in mollusks, as evidenced by the pronounced difference in the neural system. This expansion is likely an adaptation to the more complex requirements of neural signal transmission. In the *Cg*NMDAR subfamily of oysters, a singular *Cg*GRIN1 with the CaM_bdg_C0 domain has been discerned. Contrarily, zebrafish exhibit an array of GRIN copies, each embedded with the CaM_bdg_C0 domain, pivotal for the regulation of Ca^2+^ influx (Cox et al., 2005). Such distinctions underscore the potential evolutionary adaptation of calcium ion mediation in tandem with the intricacies of the neural system. In summary, the genomic variations in iGluRs family genes between oysters and vertebrates underscore the evolutionary intricacies and adaptive nature of neural systems, especially in calcium ion mediation, to environmental complexity.

In both vertebrate and invertebrate species, proteins of the iGluRs class, including those in the *Cg*iGluRs family, consistently exhibit four distinct transmembrane structural domains. Intriguingly, comparative analyses reveal no significant divergence in these domains across the various iGluRs subfamilies. Within vertebrates, these subfamilies are systematically designated based on their specific affinities for synthetic agonists, namely, AMPA, NMDA, and kainate (Hansen et al., 2021). In *Cg*GRIN1, the NMDA receptor GRIN1 subunit calmodulin binding domain C0 domain (CaM_bdg_C0) was found. This is a necessary subunit that allows Ca^2+^ to pass through, constant with the role of GRIN1 as an essential subunit of the NMDA receptor and its mediating function of Ca^2+^ channels (Ganor and Levite, 2014). There was an ANF_receptor domain identified in *Cg*GRIN2A and *Cg*GRIK2-like. The rest of the *Cg*iGluRs possess the PBP_Type_2 superfamily domain. These domains form the structural basis for the C-terminal structural domain (CTD) of *Cg*iGluRs to recognize extracellular signals. In *Cg*iGluRs, the CTD length varies between different iGluRs subfamilies. Compared with *Cg*AMPAR and *Cg*KAR, *Cg*NMDA receptors, excluding GRIN1, have a longer CTD. Studies have shown that the CTD of GluN2 is the longest (Dravid et al., 2010). This diversity of subunits in the CTD is thought to play specialized and complex roles in neurons. The above results indicate that *Cg*iGluRs share similar domain structure with their homologues from other species, and the structural differences between members of the *Cg*iGluRs family may directly reflect their functional diversity.

Phylogenetic analysis showcases a primary bifurcation of the *Cg*iGluRs family into NMDA-type and non-NMDA-type receptors, aligning with prior research (Stroebel and Paoletti, 2021). *Cg*GRIA4, *Cg*GRIK3, and *Cg*GRIK5 cluster with other invertebrate iGluRs proteins like the GRIK2 and GRIK3 of the Portuguese oyster, and the GRIK2 of the California double sheath, highlighting a close phylogenetic relationship. This relationship is likely fostered by structural similarities between AMPA-type and KA-type receptors, and is further supported by the molecular secretion complexity of glutamatergic synapses, illustrating an intricate evolutionary interplay. A comparative study reveals a significant phylogenetic link between the iGluRs families of the Pacific oyster and the California Sea Hare (*Aplysia californica*), with bootstrap analyses supporting this relationship. This underlines the conservation of the iGluRs family across species and its key role in environmental adaptability. NMDAR subfamily members are found from bacteria to mammals, suggesting it as the most ancestral lineage, followed by KAR and AMPAR (Chen et al., 1999). The iGluRs family attains functional diversity via subunit combinations and RNA editing, vital for environmental adaptation. The divergence into NMDA and non-NMDA types might reflect environmental pressures, with each type potentially offering different adaptive advantages in response to varying environmental conditions such as temperature and salinity changes (Busnardo et al., 2016; Stroebel and Paoletti, 2021). This insight offers a refined perspective on the role of iGluRs in neural signal transmission and environmental adaptability.

Prior research underscores the crucial role of iGluRs in embryonic development, with AMPA (3, 4) and Kainate (3, 4, 5) receptor abnormalities affecting mouse blastocyst development (Spirkova et al., 2022). While in vertebrates like mice, iGluRs function primarily as excitatory neurotransmitters, in bivalves, they serve different functional roles, illuminating the functional divergence across phylogenetically distant taxa. This study reveals an increase in iGluRs expression correlating with oyster larvae development (egg average RPKM = 1.5, juvenile average RPKM = 137.9), indicating *Cg*iGluRs’ involvement in this process. Notably, iGluRs expression escalates during the pediveliger period (average RPKM = 133.7), a critical stage where the eyespot and the foot develop. Recent findings suggest the eyespot has photoreceptive abilities, and the foot engages in sensory perception and locomotion (Vogeler et al., 2016; Zhang et al., 2021). Vertebrate iGluRs are pivotal in signal transduction for environmental cue perception (Levitz et al., 2016; van Giesen and Garrity, 2017). The pronounced iGluRs expression during the pediveliger stage highlights their essential role in pediveligers’ environmental perception. Particularly, *Cg*GRIA4 expression peaks in this phase (RPKM = 416), aligning with its AMPA-type counterparts. The functional assembly of AMPA-type receptors as either homomeric or heteromeric tetramers (Hanada, 2020) suggests the dominance of CgGRIA4 in steering the perceptual processes of pediveligers.

iGluRs are central to neural systems, mediating complex cerebral functions including neural transmission and memory (Sachser et al., 2017; Shahin et al., 2018; Hayashi, 2021). Our data from adult oysters show receptor-specific expression profiles. Elevated expression of *Cg*GRIA4 in the mantle suggests its role in sensory and environmental perception, aligning with GRIA4’s known role in vertebrate synaptic transmission (Sagata et al., 2010). Enhanced *Cg*GRIK1 expression in the labial palps hints at its potential role in alimentary or environmental detection, mirroring the sensory function of GRIK1 (Englund et al., 2021). Notably, pronounced *Cg*GRIN2A expression in the adductor muscle indicates possible implications in shell dynamics, resonating with Zhao et al. (2023) identification of GRIN2A as a neural excitability modulator. Our findings highlight the critical roles of *Cg*iGluRs in oyster physiology, emphasizing their evolutionary conservation and parallels with vertebrate neural systems. Additionally, we propose that oyster iGluRs might detect metal concentration changes, with certain metal ions potentially interacting with specific iGluRs domains, thus altering channel dynamics, and influencing neural or other physiological responses to environmental stress.

iGluRs are instrumental in mitigating the neurotoxic effects of heavy metals (Slotkin and Seidler, 2009). Our analyses delineate the nuanced responses of *Cg*iGluRs to specific metal challenges. Intriguingly, *Cg*GRIA1 expression is reduced under acute exposure to five metals (Zn, Cu, Cd, Hg, and Pb), yet demonstrates resilience during extended Cu and Pb challenges. This pattern suggests that *Cg*GRIA1 might bolster cellular robustness by dynamically modulating its expression in response to metal-induced stress. Studies in vertebrates emphasize the neuroprotective advantages of GRIA1 downregulation. Furthermore, shifts in the GRIA1 to GRIA2 ratio are postulated to modulate the calcium permeability of CP-AMPAR (Li et al., 2023). In our dataset, *Cg*GRIA2 shows a contrasting expression pattern under copper and lead exposure. Given GRIA2’s pivotal role in dictating AMPAR calcium permeability, it is plausible that *Cg*AMPAR receptors adapt to metal stress by fine-tuning calcium homeostasis. We observed a marked upregulation of *Cg*GRIA4 under metal stress. As a subtype of the AMPA receptor, GRIA4 is integral to rapid synaptic signaling (Song and Huganir, 2002). The pronounced expression of *Cg*GRIA4 intimates an adaptive strategy in oysters, potentially fortifying cellular defenses against metal-induced stress. However, while the majority of AMPA receptors typically have low calcium permeability, certain stressors, such as metals, might amplify this characteristic, risking neuronal integrity. The enhanced expression of *Cg*GRIA4, albeit potentially beneficial, could also precipitate calcium dysregulation and subsequent neurotoxicity if unchecked (Kim and von Gersdorff, 2016; Yang et al., 2023).

Within the neural framework, iGluRs play an indispensable role in preserving neuronal health and orchestrating functional dynamics. Our research highlights the sensitivity of *Cg*GRIA4 to heavy metal stress. We observed genes with expression patterns that mirror *Cg*GRIA4, primarily associated with Glutamatergic and Dopaminergic synapses. GRIA4, recognized for its prompt responsiveness to glutamate, is pivotal in facilitating efficient neurotransmission (Tritsch and Sabatini, 2012). Parallelly, dopaminergic modulation has been documented to sculpt the functional dynamics and membrane transport of AMPA receptors. This interplay suggests that *Cg*GRIA4 might channel neurotransmission via the Glutamatergic pathway, with its signal intensity potentially under the regulatory purview of the Dopaminergic signaling axis. Additionally, our data suggest a potential role for *Cg*GRIA4 in pathways related to neurodegenerative conditions. The activation of these pathways appears to be intertwined with perturbations in calcium homeostasis (Wakazono et al., 2023), underscoring the prospective role of *Cg*GRIA4 in bolstering cellular defenses against heavy metal stress through judicious calcium regulation. Additionally, our analysis reveals a pronounced enrichment in antioxidant metabolic pathways, notably those pivoting around Vitamin B6 and selenium derivatives (Binte Hossain et al., 2018; Ko et al., 2022). This enrichment suggests a strategic role for *Cg*GRIA4 in mitigating oxidative duress stemming from heavy metal exposure, echoing seminal research that underscores the neuroprotective virtues of Vitamin B6 and selenium compounds in countering oxidative stress. This suggests a strategic role for *Cg*GRIA4 in mitigating oxidative stress from heavy metal exposure, possibly aiding in oysters’ environmental adaptability to varying conditions like different water temperatures, salinity levels, or pollution levels.

## 5 Conclusion

This research illuminates the pervasive distribution of iGluRs in oysters, emphasizing their central importance in physiological functions. A comprehensive set of iGluRs family genes has been identified within the genome, with these genes demonstrating varied expression patterns across developmental stages, within adult tissues, and under the duress of heavy metal stress. Importantly, our findings indicate that *Cg*GRIA4 can actively respond to heavy metal stress, potentially aiding cells in resisting such stress by engaging in neural signal transmission and antioxidant stress response. However, the specific modulation of these signaling pathways by *Cg*GRIA4, and the question of whether its upregulation might precipitate calcium overload and neurotoxicity, still necessitates further exploration. These findings provide a fresh vantage point for a more profound understanding of the mechanisms of neurotoxicity.

## Data Availability

The original contributions presented in the study are publicly available. This data can be found here: https://www.ncbi.nlm.nih.gov/bioproject/PRJNA146329.

## Footnotes

1
https://www.ncbi.nlm.nih.gov/

2
https://www.uniprot.org/

3
https://www.ncbi.nlm.nih.gov/orffinder/

4
http://smart.embl-heidelberg.de/

5
https://meme-suite.org/meme/tools/meme

6
https://web.expasy.org/compute_pi/

7
https://www.geneious.com/

8
http://www.atgc-montpellier.fr/

9
http://tree.bio.ed.ac.uk/software/Figtree/

10
https://jvenn.toulouse.inrae.fr/app/example.html

11
https://www.genedenovo.com/

## Supplementary material

SUPPLEMENTARY TABLE S1 Statistical information on the amino acid sequences of the iGluRs of the selected species (search databases: NCBI, Uniprot).

SUPPLEMENTARY TABLE S2 Statistical table of KEGG-enriched gene and pathway information.

SUPPLEMENTARY FIGURE S1 Phylogenetic relationships and protein motifs of vertebrates iGluRs genes (*Homo sapiens*, *Mus musculus*, *Danio rerio*). (A) Phylogenetic tree of vertebrates iGluRs (*Homo sapiens*, *Mus musculus*, *Danio rerio*). Protein sequences were aligned using AliView, and the phylogenetic tree was constructed applying the maximum likelihood method. (B) Protein motifs of vertebrates iGluRs (*Homo sapiens*, *Mus musculus*, *Danio rerio*). Conserved motifs (1–12) are depicted by different colored boxes, with non-conserved sequences represented by black lines. Motifs were visualized using Tbtools.

SUPPLEMENTARY FIGURE S2 Phylogenetic tree and gene structure of *Cg*iGluRs. (A) Phylogenetic tree of *Cg*iGluRs. (B) Black lines, green and yellow boxes indicate the structure of the untranslated region (UTR), Intervening region, and expressed region.

SUPPLEMENTARY FIGURE S3 Temporal trend and clustering of gene expression under different metal stimulation were analyzed using the Mfuzz package. A to E correspond to different time patterns of protein expression under the stimulation of five metals (Cd, Cu, Hg, Pb, and Zn). The x axis represents three stimulation duration, while the y axis represents log2-transformed, normalized intensity ratios in each stage.
