# Supplementary material for: Unveiling the functional diversity of ionotropic glutamate receptors in the Pacific oyster (Crassostrea gigas) by systematic studies
Source: Front Physiol. 2023 Oct 25;14:1280553. doi: 10.3389/fphys.2023.1280553 (PMC10642201; doi:10.3389/fphys.2023.1280553)
Supplement: Supplementary file 1 [file DataSheet1.pdf]

Table S1 Statistical information on the amino acid sequences of the iGluRs of the selected species (search databases: NCBI, Uniprot).

Species Name	Abbreviation	Latin name	Protein ID (Uniprot/NCBI)	Gene Name
Human	H.SA	*Homo sapiens*	sp|Q05586|	GRIN1
Human	H.SA	*Homo sapiens*	sp|Q12879|	GRIN2A
Human	H.SA	*Homo sapiens*	sp|Q13224|	GRIN2B
Human	H.SA	*Homo sapiens*	sp|Q14957|	GRIN2C
Human	H.SA	*Homo sapiens*	sp|O15399|	GRIN2D
Human	H.SA	*Homo sapiens*	sp|Q8TCU5|	GRIN3A
Human	H.SA	*Homo sapiens*	sp|O60391|	GRIN3B
Human	H.SA	*Homo sapiens*	sp|P42261|	GRIA1
Human	H.SA	*Homo sapiens*	sp|P42262|	GRIA2
Human	H.SA	*Homo sapiens*	sp|P42263|	GRIA3
Human	H.SA	*Homo sapiens*	sp|P48058|	GRIA4
Human	H.SA	*Homo sapiens*	sp|P39086|	GRIK1
Human	H.SA	*Homo sapiens*	sp|Q13002|	GRIK2
Human	H.SA	*Homo sapiens*	sp|Q13003|	GRIK3
Human	H.SA	*Homo sapiens*	sp|Q16099|	GRIK4
Human	H.SA	*Homo sapiens*	sp|Q16478|	GRIK5
Chicken	G.GA	*Gallus gallus*	tr|Q6R6I2|	GRIN1
Chicken	G.GA	*Gallus gallus*	tr|A0A8V0Z475|	GRIN2A
Chicken	G.GA	*Gallus gallus*	tr|F1NMB0|	GRIN2B
Chicken	G.GA	*Gallus gallus*	tr|A0A8V1AEJ9|	GRIN2C
Chicken	G.GA	*Gallus gallus*	tr|A0A8V0XS88|	GRIN3A
Chicken	G.GA	*Gallus gallus*	tr|A0A8V1ABD2|	GRIN3B
Chicken	G.GA	*Gallus gallus*	tr|Q90855|	GRIA1
Chicken	G.GA	*Gallus gallus*	tr|A0A8V0Y5C3|	GRIA2
Chicken	G.GA	*Gallus gallus*	tr|A0A8V0Y8I2|	GRIA3
Chicken	G.GA	*Gallus gallus*	tr|A0A1D5PJ69|	GRIA4
Chicken	G.GA	*Gallus gallus*	tr|A0A8V0YIT1|	GRIK1
Chicken	G.GA	*Gallus gallus*	tr|F1NU20|	GRIK2
Chicken	G.GA	*Gallus gallus*	tr|F1P0L4|	GRIK3
Chicken	G.GA	*Gallus gallus*	tr|E1C2Q1|	GRIK4

Frog	X.TR	*Xenopus tropicalis*	tr|A0A803KAR9|	GRIN1
Frog	X.TR	*Xenopus tropicalis*	tr|F6X9P6|	GRIN2A
Frog	X.TR	*Xenopus tropicalis*	tr|A0A6I8QFJ2|	GRIN2B
Frog	X.TR	*Xenopus tropicalis*	tr|F6YJN8|	GRIN2C
Frog	X.TR	*Xenopus tropicalis*	tr|A0A6I8T1M1|	GRIN2D
Frog	X.TR	*Xenopus tropicalis*	tr|A0A803JYM2|	GRIN3A
Frog	X.TR	*Xenopus tropicalis*	tr|A0A6I8PVN6|	GRIN3B
Frog	X.TR	*Xenopus tropicalis*	tr|A0A6I8RME3|	GRIA1
Frog	X.TR	*Xenopus tropicalis*	tr|A0A8J1J504|	GRIA2
Frog	X.TR	*Xenopus tropicalis*	tr|A0A8J0SK19|	GRIA3
Frog	X.TR	*Xenopus tropicalis*	tr|A0A6I8S916|	GRIA4
Frog	X.TR	*Xenopus tropicalis*	tr|A0A6I8RQ53|	GRIK1
Frog	X.TR	*Xenopus tropicalis*	tr|A0A6I8SEA9|	GRIK2
Frog	X.TR	*Xenopus tropicalis*	tr|A0A6I8RI88|	GRIK3
Frog	X.TR	*Xenopus tropicalis*	tr|A0A6I8RC42|	GRIK4
Frog	X.TR	*Xenopus tropicalis*	tr|A0A6I8RZM4|	GRIK5
Zebrafish	D.RE	*Danio rerio*	tr|F1R366|	GRIN1a
Zebrafish	D.RE	*Danio rerio*	tr|Q6ZM67|	GRIN1b
Zebrafish	D.RE	*Danio rerio*	tr|F1QDE5|	GRIN2A
Zebrafish	D.RE	*Danio rerio*	tr|A2BH14|	GRIN2B
Zebrafish	D.RE	*Danio rerio*	tr|E7FGL8|	GRIN2C
Zebrafish	D.RE	*Danio rerio*	tr|I3NI77|	GRIN2D
Zebrafish	D.RE	*Danio rerio*	tr|A0A8M3AWH5|	GRIN3A
Zebrafish	D.RE	*Danio rerio*	tr|A0A8M9P9F6|	GRIN3B
Zebrafish	D.RE	*Danio rerio*	tr|E7F1V8|	GRIA1

Zebrafish	D.RE	*Danio rerio*	tr|A0A8M2BI34|	GRIA2
Zebrafish	D.RE	*Danio rerio*	tr|Q71E61|	GRIA3
Zebrafish	D.RE	*Danio rerio*	tr|Q71E59|	GRIA4
Zebrafish	D.RE	*Danio rerio*	XP_009303594.1	GRIK1
Zebrafish	D.RE	*Danio rerio*	sp|A0A2R8QF68|	GRIK2
Zebrafish	D.RE	*Danio rerio*	XP_017206939.1	GRIK3
Zebrafish	D.RE	*Danio rerio*	XP_021322087.1	GRIK4
Zebrafish	D.RE	*Danio rerio*	XP_009290380.1	GRIK5
Ctyela Clava	C.IN	*Ciona intestinalis*	tr|A0A1Q2TSX5|	GRIA_V1
Ctyela Clava	C.IN	*Ciona intestinalis*	tr|A0A1Q2TSV5|	GRIA_V2
Ctyela Clava	C.IN	*Ciona intestinalis*	XP_026694371.1	GRIK2
Ctyela Clava	C.IN	*Ciona intestinalis*	XP_009860945.2	GRIN1
Ctyela Clava	C.IN	*Ciona intestinalis*	XP_009859789.2	GRIN2A
Ctyela Clava	C.IN	*Ciona intestinalis*	XP_009858118.2	GRIA2
Octopus	O.BI	*Octopus bimaculoides*	XP_014790760.1	GRIN1
Octopus	O.BI	*Octopus bimaculoides*	XP_014768777.1	GRIK2
Octopus	O.BI	*Octopus bimaculoides*	XP_052828388.1	GRIN3A
Octopus	O.BI	*Octopus bimaculoides*	XP_052821854.1	GRIN2A
Ovula ovum	A.CA	*Aplysia californica*	XP_005107294.2	GRIK4
Ovula ovum	A.CA	*Aplysia californica*	XP_012935403.2	GRIK5
Ovula ovum	A.CA	*Aplysia californica*	XP_012936818.1	GRIK2
Ovula ovum	A.CA	*Aplysia californica*	AAP80570.1	GRIN
Ovula ovum	A.CA	*Aplysia californica*	NP_001191609.1	GRIA2

Lingula	L.AN	*Lingula anatina*	XP_013416499.1	GRIN1
Lingula	L.AN	*Lingula anatina*	XP_013407964.2	GRIK2
Lingula	L.AN	*Lingula anatina*	XP_013398374.1	GRIN2B
Yellow croaker	L.CR	*Larimichthys crocea*	TMS08488.1	GRIN1
Yellow croaker	L.CR	*Larimichthys crocea*	KAE8283010.1	GRIN2A
Yellow croaker	L.CR	*Larimichthys crocea*	KAE8282892.1	GRIN2B
Yellow croaker	L.CR	*Larimichthys crocea*	KAE8282756.1	GRIN2C
Yellow croaker	L.CR	*Larimichthys crocea*	KAE8288903.1	GRIN2D
Yellow croaker	L.CR	*Larimichthys crocea*	TMS00972.1	GRIN3A
Yellow croaker	L.CR	*Larimichthys crocea*	KAE8290161.1	GRIN3B
Yellow croaker	L.CR	*Larimichthys crocea*	KAE8286175.1	GRIA1
Yellow croaker	L.CR	*Larimichthys crocea*	TMS17303.1	GRIA2
Yellow croaker	L.CR	*Larimichthys crocea*	TMS14435.1	GRIA3
Yellow croaker	L.CR	*Larimichthys crocea*	KAE8295399.1	GRIA4
Yellow croaker	L.CR	*Larimichthys crocea*	XP_010735321.1	GRIK1
Yellow croaker	L.CR	*Larimichthys crocea*	TMS12660.1	GRIK2
Yellow croaker	L.CR	*Larimichthys crocea*	XP_010729958.1	GRIK3
Yellow croaker	L.CR	*Larimichthys crocea*	TMS07739.1	GRIK4
Yellow croaker	L.CR	*Larimichthys crocea*	TMS10984.1	GRIK5
oyster	C.VI	*Crassostrea virginica*	XP_022340707.1	GRIK2
oyster	C.VI	*Crassostrea virginica*	XP_022340691.1	GRIK3
oyster	C.VI	*Crassostrea virginica*	XP_022317552.1	GRIN2B

oyster	C.VI	*Crassostrea virginica*	XP_022317276.1	GRIN3A
oyster	C.VI	*Crassostrea virginica*	XP_022290701.1	GRIN1

Table S2 Statistical table of KEGG-enriched gene and pathway information.

Pathway ID	Gene ID	Gene name	Full name
ko05033	XP_011426146.2	GRIA4	glutamate receptor 4
ko05017	XP_011426146.2	GRIA4	glutamate receptor 4
ko04730	XP_011426146.2	GRIA4	glutamate receptor 4
ko05016	XP_011426146.2	GRIA4	glutamate receptor 4
ko05031	XP_011426146.2	GRIA4	glutamate receptor 4
ko05202	XP_011426146.2	GRIA4	glutamate receptor 4
ko04723	XP_011426146.2	GRIA4	glutamate receptor 4
ko04724	XP_011426146.2	GRIA4	glutamate receptor 4
ko04728	XP_011426146.2	GRIA4	glutamate receptor 4
ko04080	XP_011426146.2	GRIA4	glutamate receptor 4
ko04713	XP_011426146.2	GRIA4	glutamate receptor 4
ko04024	XP_011426146.2	GRIA4	glutamate receptor 4
ko05033	XP_011426147.3	GRIA4	glutamate receptor 4
ko05017	XP_011426147.3	GRIA4	glutamate receptor 4
ko04730	XP_011426147.3	GRIA4	glutamate receptor 4
ko05016	XP_011426147.3	GRIA4	glutamate receptor 4
ko05031	XP_011426147.3	GRIA4	glutamate receptor 4
ko05202	XP_011426147.3	GRIA4	glutamate receptor 4
ko04723	XP_011426147.3	GRIA4	glutamate receptor 4
ko04724	XP_011426147.3	GRIA4	glutamate receptor 4
ko04728	XP_011426147.3	GRIA4	glutamate receptor 4
ko04080	XP_011426147.3	GRIA4	glutamate receptor 4
ko04713	XP_011426147.3	GRIA4	glutamate receptor 4
ko04024	XP_011426147.3	GRIA4	glutamate receptor 4
ko05033	XP_011426148.2	GRIA4	glutamate receptor 4
ko05017	XP_011426148.2	GRIA4	glutamate receptor 4
ko04730	XP_011426148.2	GRIA4	glutamate receptor 4
ko05016	XP_011426148.2	GRIA4	glutamate receptor 4
ko05031	XP_011426148.2	GRIA4	glutamate receptor 4
ko05202	XP_011426148.2	GRIA4	glutamate receptor 4
ko04723	XP_011426148.2	GRIA4	glutamate receptor 4
ko04724	XP_011426148.2	GRIA4	glutamate receptor 4
ko04728	XP_011426148.2	GRIA4	glutamate receptor 4
ko04080	XP_011426148.2	GRIA4	glutamate receptor 4

ko04713	XP_011426148.2	GRIA4	glutamate receptor 4
ko04024	XP_011426148.2	GRIA4	glutamate receptor 4
ko05033	XP_011426149.2	GRIA4	glutamate receptor 4
ko05017	XP_011426149.2	GRIA4	glutamate receptor 4
ko04730	XP_011426149.2	GRIA4	glutamate receptor 4
ko05016	XP_011426149.2	GRIA4	glutamate receptor 4
ko05031	XP_011426149.2	GRIA4	glutamate receptor 4
ko05202	XP_011426149.2	GRIA4	glutamate receptor 4
ko04723	XP_011426149.2	GRIA4	glutamate receptor 4
ko04724	XP_011426149.2	GRIA4	glutamate receptor 4
ko04728	XP_011426149.2	GRIA4	glutamate receptor 4
ko04080	XP_011426149.2	GRIA4	glutamate receptor 4
ko04713	XP_011426149.2	GRIA4	glutamate receptor 4
ko04024	XP_011426149.2	GRIA4	glutamate receptor 4
ko05033	XP_011426150.2	GRIA4	glutamate receptor 4
ko05017	XP_011426150.2	GRIA4	glutamate receptor 4
ko04730	XP_011426150.2	GRIA4	glutamate receptor 4
ko05016	XP_011426150.2	GRIA4	glutamate receptor 4
ko05031	XP_011426150.2	GRIA4	glutamate receptor 4
ko05202	XP_011426150.2	GRIA4	glutamate receptor 4
ko04723	XP_011426150.2	GRIA4	glutamate receptor 4
ko04724	XP_011426150.2	GRIA4	glutamate receptor 4
ko04728	XP_011426150.2	GRIA4	glutamate receptor 4
ko04080	XP_011426150.2	GRIA4	glutamate receptor 4
ko04713	XP_011426150.2	GRIA4	glutamate receptor 4
ko04024	XP_011426150.2	GRIA4	glutamate receptor 4
ko05033	XP_011450862.2	ACHA	acetylcholine receptor subunit alpha-1-B
ko04080	XP_011450862.2	ACHA	acetylcholine receptor subunit alpha-1-B
ko05033	XP_011450863.2	ACHA	acetylcholine receptor subunit alpha-1-B
ko04080	XP_011450863.2	ACHA	acetylcholine receptor subunit alpha-1-B
ko05033	XP_019922429.2	GRIA4	glutamate receptor 4
ko05017	XP_019922429.2	GRIA4	glutamate receptor 4
ko04730	XP_019922429.2	GRIA4	glutamate receptor 4
ko05016	XP_019922429.2	GRIA4	glutamate receptor 4
ko05031	XP_019922429.2	GRIA4	glutamate receptor 4
ko05202	XP_019922429.2	GRIA4	glutamate receptor 4
ko04723	XP_019922429.2	GRIA4	glutamate receptor 4
ko04724	XP_019922429.2	GRIA4	glutamate receptor 4
ko04728	XP_019922429.2	GRIA4	glutamate receptor 4

ko04080	XP_019922429.2	GRIA4	glutamate receptor 4
ko04713	XP_019922429.2	GRIA4	glutamate receptor 4
ko04024	XP_019922429.2	GRIA4	glutamate receptor 4
ko05033	XP_019929418.2	ACHA	acetylcholine receptor subunit alpha-1-B
ko04080	XP_019929418.2	ACHA	acetylcholine receptor subunit alpha-1-B
ko05033	XP_034302102.1	GRIA4	glutamate receptor 4
ko05017	XP_034302102.1	GRIA4	glutamate receptor 4
ko04730	XP_034302102.1	GRIA4	glutamate receptor 4
ko05016	XP_034302102.1	GRIA4	glutamate receptor 4
ko05031	XP_034302102.1	GRIA4	glutamate receptor 4
ko05202	XP_034302102.1	GRIA4	glutamate receptor 4
ko04723	XP_034302102.1	GRIA4	glutamate receptor 4
ko04724	XP_034302102.1	GRIA4	glutamate receptor 4
ko04728	XP_034302102.1	GRIA4	glutamate receptor 4
ko04080	XP_034302102.1	GRIA4	glutamate receptor 4
ko04713	XP_034302102.1	GRIA4	glutamate receptor 4
ko04024	XP_034302102.1	GRIA4	glutamate receptor 4
ko05033	XP_034337064.1	ACHA	acetylcholine receptor subunit alpha-1-B
ko04080	XP_034337064.1	ACHA	acetylcholine receptor subunit alpha-1-B
ko05017	XP_011450103.1	cyt c	cytochrome c
ko05016	XP_011450103.1	cyt c	cytochrome c
ko05017	XP_011453863.2	NFYA	nuclear transcription factor Y subunit alpha
ko05017	XP_011453864.2	NFYA	nuclear transcription factor Y subunit alpha
ko05017	XP_011453865.2	NFYA	nuclear transcription factor Y subunit alpha
ko05017	XP_011453866.2	NFYA	nuclear transcription factor Y subunit alpha
ko05016	XP_011441947.1	POLR2J	DNA-directed RNA polymerase II subunit RPB11-a
ko05016	XP_034317742.1	P3A2	DNA-binding protein P3A2-like isoform X2
ko05016	XP_034317743.1	P3A2	DNA-binding protein P3A2-like isoform X2
ko05016	XP_034317744.1	P3A2	DNA-binding protein P3A2-like isoform X2

ko05016	XP_034317745.1	P3A2	DNA-binding protein P3A2-like isoform X2
ko05016	XP_034317746.1	P3A2	DNA-binding protein P3A2-like isoform X2
ko05031	XP_011424397.1	PPP2R1A	serine/threonine-protein phosphatase alpha-2 isoform
ko04728	XP_011424397.1	PPP2R1A	serine/threonine-protein phosphatase alpha-2 isoform
ko04024	XP_011424397.1	PPP2R1A	serine/threonine-protein phosphatase alpha-2 isoform
ko05202	XP_011441199.2	MYC	transcriptional regulator Myc-A
ko05202	XP_011447558.2	TFEC	transcription factor EC
ko05202	XP_011447559.2	MITF	microphthalmia-associated transcription factor
ko05202	XP_011447560.2	MITF	microphthalmia-associated transcription factor
ko04080	XP_011414282.2	P2RX4	P2X purinoceptor 4
ko04080	XP_011414283.1	P2RX4	P2X purinoceptor 4
ko04024	XP_034336923.1	RhoA	ras-like GTP-binding protein rhoA

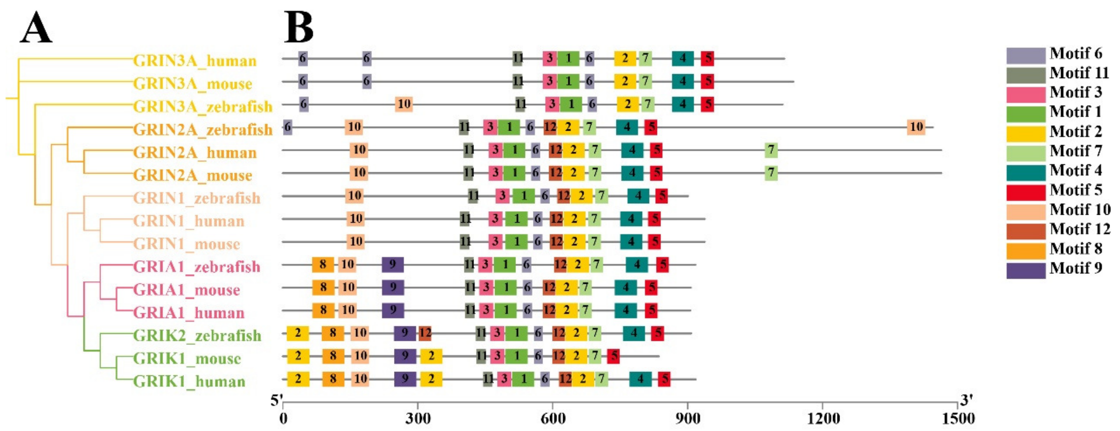

Supplementary Figure S3 Phylogenetic relationships and protein motifs of vertebrates iGluRs genes (Homo sapiens, Mus musculus, Danio rerio). (A) Phylogenetic tree of vertebrates iGluRs (Homo sapiens, Mus musculus, Danio rerio). Protein sequences were aligned using AliView, and the phylogenetic tree was constructed applying the maximumlikelihood method. (B) Protein motifs of vertebrates iGluRs (Homo sapiens, Mus musculus, Danio rerio). Conserved motifs (1–12) are depicted by different colored boxes, with non-conserved sequences represented by black lines. Motifs were visualized using Tbtools.

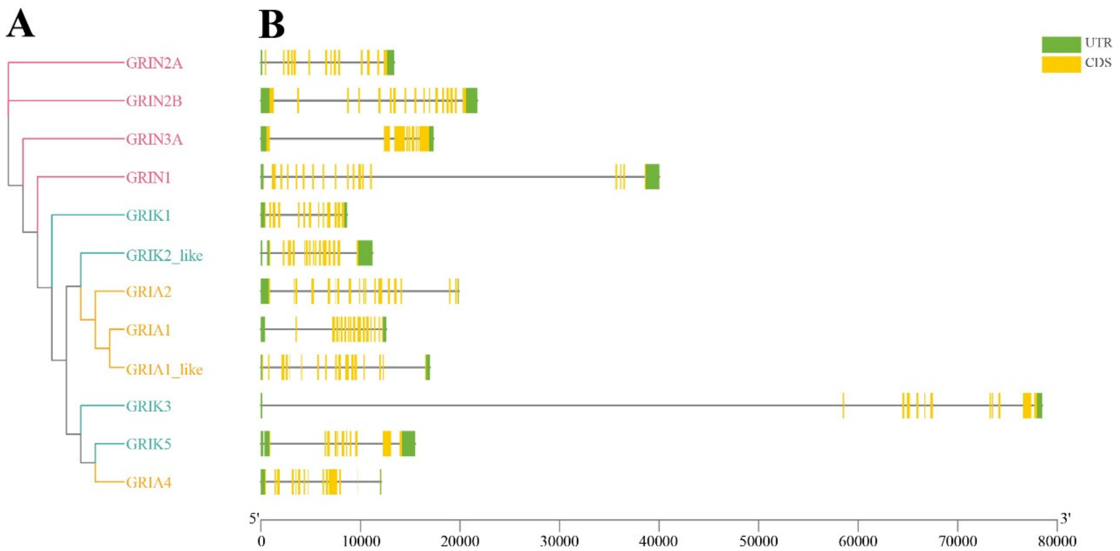

Supplementary Figure S2. Phylogenetic tree and gene structure of CgiGluRs. (A) Phylogenetic tree of CgiGluRs. (B) Black lines, green and yellow boxes indicate the structure of the untranslated region (UTR), Intervening region, and expressed region.

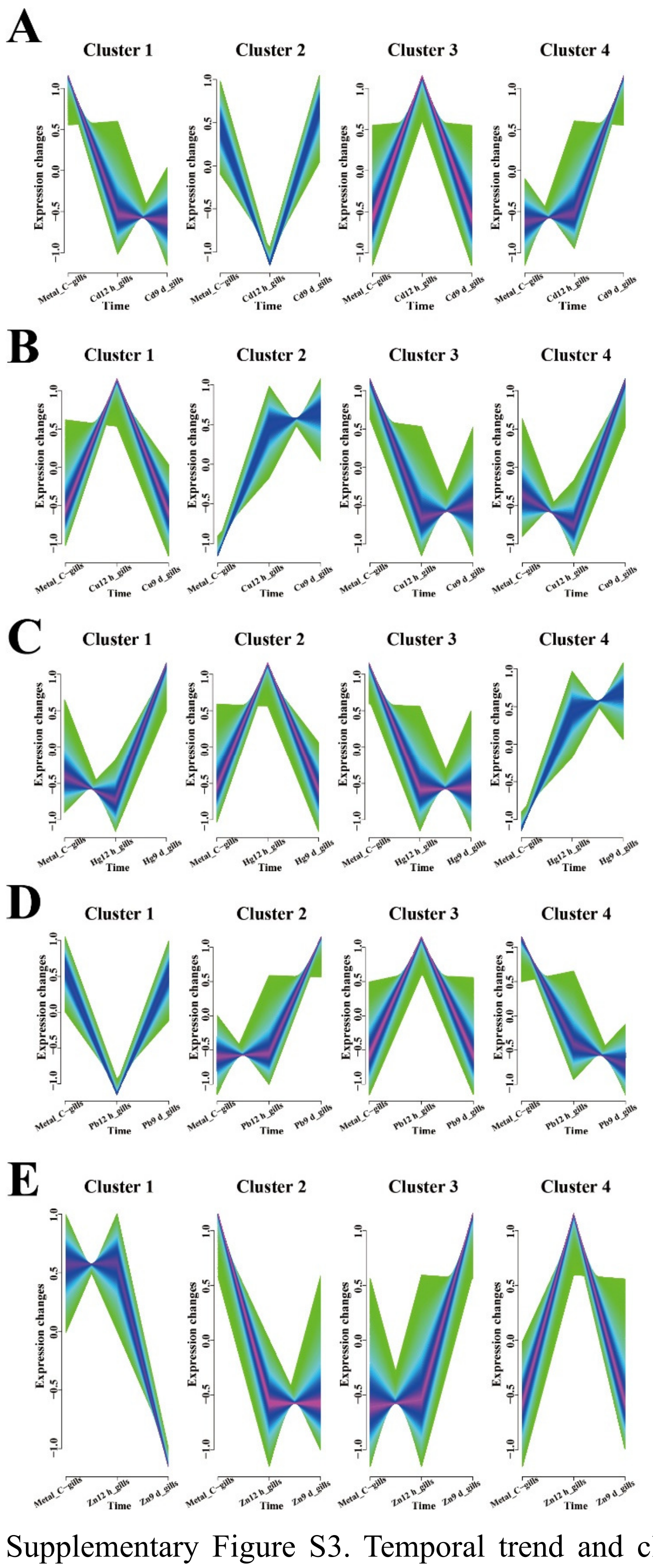

Supplementary Figure S3. Temporal trend and clustering of gene expression under different metal stimulation were analyzed using the Mfuzz package. A to E correspond to different time patterns of protein expression under the stimulation of five metals (Cd, Cu, Hg, Pb, Zn). The x axis represents three stimulation duration, while the y axis represents log2-transformed, normalized intensity ratios in each stage.
